# Nutrition self-efficacy intervention to improve nutritional status of Iranian older adults

**DOI:** 10.1186/s41043-024-00519-1

**Published:** 2024-02-02

**Authors:** Seyedeh Belin Tavakoly Sany, Hamideh Ahangari, Amir Rasoulifar, Mitra Salimi, Jamshid Jamali, Hadi Tehrani

**Affiliations:** 1 Department of Health Education and Health Promotion, School of Health, Mashhad University of Medical Sciences, Mashhad, Iran; 2 Student Research Committee, Mashhad University of Medical Sciences, Mashhad, Iran; 3 Management Education District of Mashhad, Mashhad, Iran; 4 Social Determinants of Health Research Center, Mashhad University of Medical Sciences, Mashhad, Iran; 5 Department of Biostatistics, School of Health, Mashhad University of Medical Sciences, Mashhad, Iran; 6 Department of Health, Safety and Environment, School of Health, Mashhad University of Medical Sciences, Mashhad, Iran

**Keywords:** Self-efficacy, Malnutrition, Health education, Aged, Health promotion

## Abstract

**Objectives:**

Older adults are a vulnerable group that is at risk of poor nutritional status, which can lead to disease and increase their healthcare costs. Our study aimed to investigate the impact of a self-efficacy intervention on the nutritional status of older adults.

**Methods:**

A controlled before and after study was conducted on 110 older adults in the Mashhad, Iran, from 2020 to 2022. Participants were randomly allocated to the intervention (*n *= 55) and control groups (*n *= 55). Participants in the intervention group received educational training that was based on the self-efficacy theory. The control group received the routine care. Data collection tools included demographic information questionnaire, Mini Nutritional Assessment Questionnaire, and standard self-efficacy questionnaire. The questionnaires were completed at baseline (before intervention), instantly after the intervention, and at 3-months follow-up by participants in both groups. Data were analyzed using SPSS version 25 and the significance level was considered less than 0.05.

**Results:**

The Mean of nutritional status in the intervention group, at the baseline, immediately after intervention and 3 months of follow-up were 25.1 ± 2.3, 28.3 ± 5.2 and 27.6 ± 6., respectively. This increase was significant (*p *< 0.001). Our findings revealed that self-efficacy among participants in the intervention group significantly changed (*P *< 0.001) across time from baseline through follow-up. There was no significant difference in the mean of self-efficacy and nutritional status in the control group during the study period (*P *> 0.05).

**Conclusion:**

This current study provided a basis to examine in the effectiveness of such intervention using a properly powered randomized controlled study. Therefore, it can be concluded that self-efficacy interventions are a promising approach to improving the nutritional behaviors of the older adults.

*Trial registration*: IRCT20160619028529N9.

## Introduction

The nutritional status of the elderly in West Asia, particularly in India and Saudi Arabia, presents significant challenges. In India, studies have shown that malnutrition among older adults is a prevalent problem, with rates ranging from 14.3 to 19%1 [1]. In Saudi Arabia, a cross sectional study found that 17.9% of community-dwelling older adults were malnourished, with factors such as overweight or obesity, poor oral health, and depression being associated with malnutrition. Additionally, in Southwest Ethiopia, 48.1% of elderly people were malnourished or at risk of malnutrition, with a higher prevalence in urban areas [2].

According to the World Health Organization (WHO), the number of older adults is projected to increase significantly in the coming decades [3]. Improving healthcare in recent decades and increasing life expectancy have indeed led to a growing older adults, especially in developing countries. According to the latest census conducted in 2016, about 9.2% of the total population of Iran are people aged 60 and older, which is equivalent to 7 million and 414 thousand people. This figure is expected to reach 21 to 26 million people by 2050, equivalent to 26% of the total population of Iran [4].

Older adults are at increased risk of malnutrition due to various physiological and psychological reasons. This has consequences for health, quality of life, independence, and economic conditions [5]. Proper nutrition is a critical factor in successful aging. Improper eating habits among the older adults lead to some chronic diseases such as type 2 diabetes, atherosclerosis, coronary heart disease and malnutrition. This disrupts the quality of life and leads to physical and cognitive decline [6, 7]. Reduced food intake is related to nutrient deficiencies, it leads to poor health and common problems related to aging. Various factors such as social, physical and physiological condition are related to the reduction of food consumption among the older adults, it leads to the nutrient deficiencies which indirectly associated to their poor health status [8, 9].

Improving nutrition knowledge and attitudes toward healthy eating can help individuals make better food choices and adopt healthier eating habits [6, 10]. As studies have shown, educational programs to improve the quality of nutrition using health education approaches and theories, can play an important and effective role in changing the behavior and attitudes of the older adults and improve the nutritional health of the older adults [11]. The concept of self-efficacy to explain behavioral determinants was presented for the first time by Bandura, who differentiates between efficacy expectations and outcome expectations [12]. Bandura emphasized emotional, cognitive, motivational and self-regulation processes to understand self-efficacy [13]. The importance of self-efficacy in preventive behaviors of chronic diseases such as diabetes and cardiovascular diseases in the older adults has been emphasized [14]. Salahshouri et al. [15] show a positive and significant relationship was found between self-efficacy and health-promoting behaviors such as stress management, physical activity, interpersonal social relationships and prevention. Naseh et al. [16] also observed a direct and significant correlation between self-efficacy and quality of life. Since self-efficacy can play an important role in the lives of older adults, the development of a more effective method that is widely applicable and efficient is required [14].

Considering the above, the importance of malnutrition and its consequences in the older adults and the existence of a high percentage of the older adults in Iran, it is necessary to perform educational interventions in the field of older adult’s nutrition. Among the behavioral theories, the theory of self-efficacy can provide basic information to change the attitude and nutritional behavior of the older adults, but unfortunately studies on the nutrition of the older adults using this theory were very limited, so this study aimed to determine the effect of self-efficacy intervention on the nutritional status of the older adults in Iran. This study was designed and implemented to investigating the effect of self-efficacy intervention on the nutritional status of the Iranian older adults.

## Methods

### Study design and sampling

A controlled before and after study (CBA) was conducted to evaluate the nutritional self-efficacy intervention in improving nutritional behaviors on 110 older adults who referred to primary care centers in Mashhad (Iran). In Iran, health care centers are a public sector for providing primary health care services. Primary health care is provided free of charge in these centers by health care professionals such as doctors, family health professionals, nutritionists, midwives and psychologists.

In this study, the older adults were eligible to participate in the study if (a) they had informed consent to participate in the study; (b) they did not have a record of cognitive and mental disorders (C) had no gastrointestinal or nutritional disorders and (d) were over 60 years of age. If the older adults did not cooperate to continue their studies or were absent for more than one session in the training sessions, they would be excluded from the study.

To calculate the sample size for this study, using the mean and standard deviation of the nutrition score in Vahedian Shahroodi study that is the mean and standard deviation of the difference before and after the nutrition score in the intervention and control groups were reported as 9.83 ± 9.65 and 1.50 ± 8.26, respectively [17] and considering the error of 5% and test power of 80%, the minimum sample size in each group of 44 people was determined. Due to the possibility of falling samples, the final sample size increased to 55 people in each group.

Sampling for the study began after receiving the code of ethics from the ethics committee of Mashhad University of Medical Sciences and registering the study in the Iranian clinical trial.

**Fig. 1:**
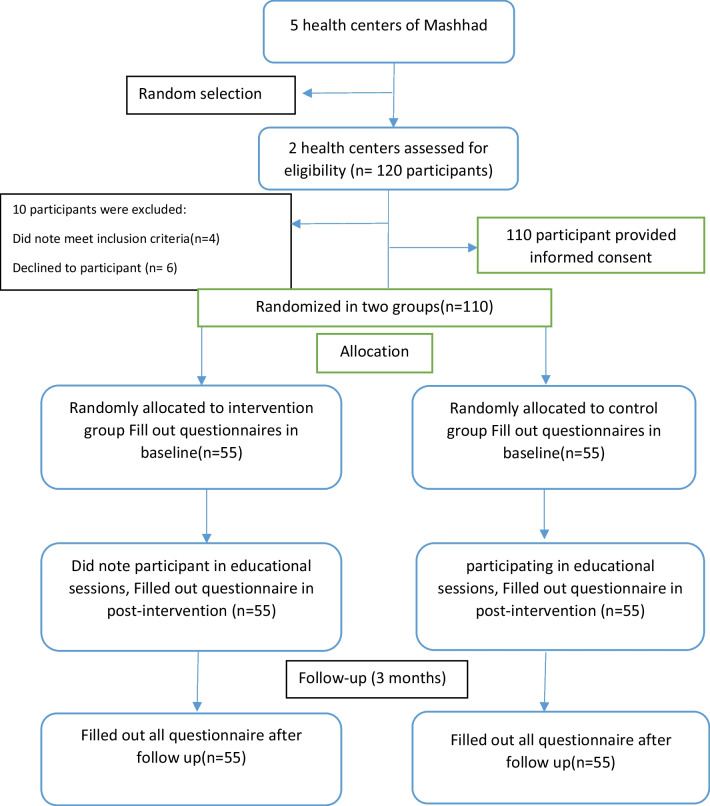
Process of selecting and tracking study participants

Out of 5 health centers in Mashhad, 2 centers were randomly selected and from each of them, 2 community health centers were selected as the intervention group and 2 community health centers, which were geographically and culturally similar to the intervention centers, were selected as the control group. The sample selection process is shown in Fig. 1.

After dividing the subjects into control and intervention groups, an educational intervention for the intervention group, based on the theory of self-efficacy with more emphasis on the factors affecting (age, sex, level of education and marriage) on the nutrition of the older adults was prepared and presented by the researcher. In order to collect information before the intervention, the questionnaires were completed by both groups and after the intervention, the questionnaires were completed again immediately and 3 months after the intervention by the by both groups.

### Data collection tools

Data collection tools included: 1—Research Inquiry Questionnaire and Survey Information Questionnaire 2—Mini Nutritional Assessment (MNA) Questionnaire and 3—Self-Efficacy Questionnaire.

1. The questionnaire used to enroll participants in the study included questions based on the criteria for inclusion and exclusion, which were determined by reviewing scientific texts. Participants answered these questions with a yes or no response. If the participants were eligible, the main questionnaires were completed and entered into the study. Survey information questionnaire also includes age, gender, education, income, height, weight, Body Mass Index [BMI, that it was calculated as weight in kilograms divided by the square of his height in meters (kg/m^2^)], and number of children.
2. To assess the nutritional status, the MNA brief nutritional review questionnaire was used. This questionnaire has been used in several studies in Iran. This questionnaire includes 4 parts related to anthropometric characteristics (body mass index, weight loss, mid-arm circumference and leg muscle environment), general characteristics (lifestyle, medications, ability to move and the presence of symptoms of depression or dementia), nutritional assessment (number Meals, food and fluid intake and eating independence) and documentary assessment (self-perception of health and nutrition). The answers to each question are awarded points. The sum of these scores classifies the individual as well-nourished (≤ 24), at nutritional risk (17–24) and malnourished (> 17). The validity and reliability of this questionnaire in the study of Mir Arefin and Partners have been approved in Iran [18]. The Mini Nutritional Assessment (MNA) has been evaluated in Iran in several studies, and the results indicate that it is a reliable and valid tool for assessing the nutritional status of the Iranian population, particularly the elderly. The Iranian version of the MNA short-form (MNA-SF) has been found to have good agreement and diagnostic accuracy [19].
3. The standard general self-efficacy questionnaire had 10 items with two separate subscales of general self-efficacy and social self-efficacy and in Iran it was standardized [20]. Cronbach's alpha coefficients was obtained 0.82 by Rajabi et al. [20]. The scoring method on this scale is as follows: 1 = not correct at all, 2 = slightly correct, 3 = somewhat correct, 4 = completely correct. A person's self-efficacy score is the sum of 10 items and the range of scores is between 10 and 40. A high score on this scale indicates higher general self-efficacy. This scale does not have a cut-off point, but according to the middle of the instrument, the subjects were divided into two categories of high and low self-efficacy.

### Intervention

The intervention was conducted based on the Template for Intervention Description and Replication checklist (Table 1). Educational intervention for the intervention group, based on the theory of self-efficacy with more emphasis on the factors affecting (age, sex, level of education and marriage) on the nutrition of the older adults was prepared and presented by the researcher, four sessions was presented to the intervention group using teaching aids such as slides, pamphlets, posters, booklets and educational CDs. Outline, educational content and method of educational intervention are given in Table 2.

**Table 1:** Template for Intervention Description and Replication checklist of the study

Items	Description
BRIEF NAME	The effect of educational intervention based on self-efficacy theory on improving the nutritional status of the older adults
WHY (Rationale of treatment)	The main purpose of this controlled before and after study was to determine whether the educational intervention could be effective on the self-efficacy model for improving nutritional behaviors
WHAT (Materials)	The intervention is informed self-efficacy model
WHAT (Procedures)	This controlled before and after study comprised intervention and control groups. The intervention group received the full educational program. During the study, no intervention was performed in the control group. Focusing on self-efficacy key structures, four training sessions were organized that were explained in Table 2
WHO PROVIDED (Profession, expertise, background, specific training)	Training is provided by specialist health educators at the health care center
HOW (modes of delivery)	In the intervention group: Four training sessions were conducted face-to-face by lectures, group discussions, answers and questions
WHERE (Infrastructure and relevant features)	Primary health care centers
WHEN and HOW MUCH (Number of sessions, duration, intensity or dose)	Each senior participates in an educational program that consists of four 90-min sessions twice a week for one month
TAILORING (Personalization)	The training sessions in the study consisted of the following components, with each session standardized in terms of time and content: question and answer, Lectures, Educational pamphlets, Focus group discussions
MODIFICATIONS	Intervention follow-up was optimal for all participants
HOW WELL: planned (Follow and procedure to preserve it)	for all participants, following the intervention was optimal
HOW WELL: actual	Without any deviation from the organized protocol, the complete planned intervention program was delivered to all participants

**Table 2:** Outline, educational content and method of educational intervention

Times	Intervention method	Intervention’s instruction
Session1	PowerPoint, lecture, rain of thoughts, group discussion and pamphlets and poster	Introduction and acquaintance with group members Familiarity with the need for proper nutrition in old age according to age The importance of having a proper body mass in the health of the older adults Nutritional differences in the older adults
Session2	PowerPoint, lecture, rain of thoughts, group discussion and pamphlets and poster	Causes of malnutrition in the older adults Risks of malnutrition and malnutrition in the older adults Proper principles of malnutrition prevention The role of nutrition in disease prevention Nutritional needs in the older adults Types of food groups (5 food groups)
Session3	Group discussion, pamphlets and posters	The role of self-efficacy programs in improving nutritional status Personal abilities in improving nutritional status Training in muscle relaxation Ways to remove barriers to nutritional improvement in old age
Session4	PowerPoint, lecture, rain of thoughts, group discussion and pamphlets and poster	Useful tips for proper nutrition Provide alternative behaviors to having a proper diet Ways to succeed in having a balanced diet

### Data analysis

Data related to demographic and contextual observations of the participants were analyzed using SPSS version 25. The Kolmogorov–Smirnov was used to test whether two distributions differ. We also conducted the descriptive analysis (frequency, mean, and standard deviation) and bivariate analyses (paired *t* test, Wilcoxon test, and Friedman) to quantify variation of different variables in intervention and control groups. *P* value of < 0.05 was considered to be statistically significant.

## Results

In this study, 110 older adults referring to Mashhad comprehensive health service centers participated, of which 55 were in the control group and 55 were in the intervention group. In this study, majority of participants were female (63.6%), married (70%), Primary and secondary education (53.6%), with an income of 10–15 million $ monthly (50%). 49.1% in the control group and 47.3% of the participants in the intervention group are in the category of 69–60 years. The results of Fisher's test showed that there was no significant difference between intervention and control groups in terms of demographic characteristics (*p *> 0.05) (Table 3).

**Table 3:** Baseline characteristics in the control and intervention groups (*n *= 110)

Characteristics		Intervention *n* (%)	Control *n* (%)	Total *n* (%)	Test result
Marital status	Single	0 (0/0)	0 (0/0)	0 (0/0)	*P *= 0.663 $${\chi }^{2}=0.82$$
	Married	40 (72.7)	37 (67.3)	77 (70)	
	Divorced	2 (3.6)	4 (7.3)	6 (5/5)	
	Widows	13 (23.6)	14 (25.5)	27 (24.5)	
Education	Illiterate	26 (47.3)	23 (41.8)	49 (44.5)	*P *= 0.578 *Z *= 0.55
	< High school	28 (50.9)	31 (56.4)	59 (53.6)	
	> High school	1 (1.8)	1 (1.8)	2 (1.8)	
Gender	Male	21 (38.2)	19 (34.5)	40 (36.4)	*P *= 0.692 $${\chi }^{2}=0.15$$
	Female	34 (61.8)	36 (65.5)	70 (63.36)	
Age	60–69	26 (47.3)	27 (49.1)	53 (48.2)	*P *= 0.822 *Z *= 0.22
	70–79	21 (38.2)	16 (29.1)	37 (33.6)	
	> 80	8 (14.5)	21 (38.2)	20 (18.2)	
BMI	25 >	19 (34.5)	(5/14) 8	40 (36.4)	*P *= 0.293 $${\chi }^{2}=2.45$$
	25 < =	36 (65.5)	34 (61.8)	70 (63.6)	
Income per month	< 10$	3 (5.5)	4 (7.3)	(6/63) 70	*P *= 0.727 *Z *= 0.34
	10$- 15$	27 (49.1)	28 (50.9)	(55 (50)	
	15$-20$	12 (21.8)	10 (18.2)	22 (20)	
	> 20$	13 (23.6)	13 (23.6)	26 (23.6)	
Number of children	0	0 (0)	0 (0)	0 (0)	*P *= 0.379 *Z *= 0.87
	1–2	6 (10.9)	5 (9.1)	11 (10)	
	3–4	39 (70.9)	36 (65.5)	75 (68.2)	
	5 >	10 (18.2)	14 (25.5)	24 (21.8)	

The results of the study comparing the mean scores of nutritional status and self-efficacy (before intervention, immediately after intervention, and 3-months follow-up) are presented in Table 4. The study found no significant difference (*P *> 0.05) between the scores nutritional status and self-efficacy in the control and intervention groups at baseline. (Table 4).

**Table 4:** Mean and standard deviation of nutritional score and self-efficacy score of the older adults in the control and test groups, before and after the educational intervention (*n *= 110)

Variables		Pre intervention (mean ± SD)	After intervention (mean ± SD)	Mean difference (mean ± SD)	‡*p* value	3-month follow-up (mean ± SD)	Mean difference (mean ± SD)	§*p* value
Nutrition	Intervention group	25.1 ± 2.3	28.3 ± 5.2	3.2 ± 1.94	0.001	27.6 ± 6.3	2.5 ± 2.24	0.001
	Control group	25.3 ± 3.3	25.4 ± 2.2	0.1 ± .02	0.448	25.2 ± 0.3	− 0.10 ± 3.2	0.568
	†*p* value	0.961*	0.001	0.001		0.001	0.001	
Self-efficacy	Intervention group	19.3 ± 4.8	22.4 ± 3.8	3.1 ± 1.4	0.001	22.7 ± 3.6	3.4 ± 2.1	0.001
	control group	19.3 ± 7.5	19.3 ± 3.7	0.0 ± 3.9	0.094	19.5 ± 3.7	0.2 ± 2.6	0.127
	†* p* value	0.626	0.001	0.001		0.001	0.001	

^†^Testing significant change between control and interventional groups

^‡^Testing significant change in interventional group pre and post intervention and control group

^§^Testing significant change in interventional group pre and 3-month follow-up and control group

The mean of nutritional status in the intervention group at the baseline was 25.1 ± 2.3. The Mean of nutritional status in the intervention group, immediately after intervention and 3 months of follow-up were 28.3 ± 5.2 and 27.6 ± 6., respectively. This increase was significant (*p *< 0.001), while in the control group, the mean nutritional status before the intervention, after the intervention and 3 months of follow-up, were 25.3 ± 3.3, 25.4 ± 2.2 and 25.2 ± 0.3, respectively (*P *> 0.05). There was no significant difference in the mean of nutritional status in the control group during the study period. (Table 4).

Our findings revealed that self-efficacy among participants in the intervention group significantly changed (*P *< 0.001) across time from baseline through follow-up. The mean of self-efficacy in the intervention group and control group at the baseline was 19.3 ± 4.8 and 19.3 ± 7.5, respectively (*P *> 0.05). The Mean of self-efficacy in the intervention group, immediately after intervention and 3 months of follow-up were 22.4 ± 3.8 and 22.7 ± 3.6, respectively. This increase was significant (*p *< 0.001) while in the control group, the mean of self-efficacy score, after the intervention and 3 months of follow-up, were 19.3 ± 3.7 and 19.5 ± 3.7, respectively (*P *> 0.05). There was no significant difference in the mean of self-efficacy scores in the control group during the study period (Table 4).

## Discussions

We conducted this study to investigate the impact of a self-efficacy intervention on the nutritional status of the Iranian older adults.

Our findings showed that in the baseline, participants had a low level of information and skills necessary for healthy eating. However, in the intervention group a significant increase in the mean score of nutrition was observed in the after intervention and follow-up. This may be due to their increased knowledge and commitment to nutritional behaviors. During the educational intervention, all older adults learned how to set realistic goals to increase their commitment and time to eat a healthy diet each day or to make small changes that could be made to have healthier diets. Therefore, most of the older adults in the intervention group have higher individual ability and self-confidence to regularly perform appropriate behaviors even in conflict situations [21]. This result was consistent with previous studies which showed that increasing people's knowledge can lead to a higher level of change and commitment to modify healthy eating behaviors [21–23]. Similarly, the researchers used educational intervention based on the health belief model and observed a significant improvement in the nutritional status of older women [23]. In another study, an educational intervention based on self-efficacy theory promoted a healthy lifestyle among women [24]. The training methods that used in this study, including face-to-face discussion, interactive lessons, video showing, group presentation and question and answer sessions can be key elements of the effectiveness of the intervention. Malnutrition is a significant concern for older adults due to a variety of physiological and psychological factors. The increased risk of malnutrition in older adults has significant implications for their health, quality of life, independence, and economic circumstances [23]. Teaching the principles of proper nutrition throughout life, especially old age, can improve the physical and mental health of all people, especially the older adults, because the positive effects of proper nutrition, to promote health, reduce risks and manage disease.

The current results showed that the educational intervention was able to increase self-efficacy in the intervention group. The success of the study in obtaining these results can be due to the implementation of program activities in the direction of four sources of self-efficacy. These four sources include performance success, surrogate experience, verbal persuasion, and emotional and physiological states. Verbal persuasion was used during the presentation of the educational intervention. Building trust and communication with participants was an important skill used. Older adults often learn from personal experience, so performance success was used as participants continued to practice healthy eating behaviors. Sharing experience with each other and referring to the taught material was one of the components of social and symbolic modeling in proxy experience. Older people can develop their skills with their peers [25]. Previous intervention studies have shown similar findings and reported improvement in self-efficacy scores before and after the implementation of the intervention program [26, 27]. This finding indicates the effectiveness of educational intervention on individuals to increase self-efficacy and ultimately achieve healthy behavior. Self-efficacy is defined as people's beliefs about their ability to deliver levels of performance that affect their life events. Self-efficacy beliefs determine how people feel, think, motivate, and behave. People with higher self-efficacy set higher goals and become more committed, resulting in better behavior; while, people with lower self-efficacy do not engage in appropriate behavioral outcomes. Self-efficacy determines how people assess barriers People with low self-efficacy are easily persuaded in the face of problems that their behavior is useless and give up quickly while people with high self-efficacy remove obstacles by improving self-management skills and perseverance and stand up to problems [28]. The results of the educational intervention based on the theory of self-efficacy do not crystallize immediately in the individual and it takes time to be internalized in the individual, which the results of the present study also confirm.

### Study strengths

This study is one of the first studies that evaluates the effectiveness of nutrition Self-Efficacy intervention on improving self-efficacy and nutrition status among the older adults. The use and application of theoretical theory in different populations is important in advancing theory and understanding the vital components of successful interventions. One of the unique aspects of the current study was that all the positive effects of the intervention were preserved in the 3-month follow-up. This success may be related to the constructs of self-efficacy theory, or it may be due to specific behavior change methods such as goal setting or group discussions. This finding provides a basis for future educational interventions aimed at promoting healthy behaviors. In addition, this research is possible and very acceptable because it requires minimal investment and time by training health care providers in health centers.

### Research limitations

One of the limitations of this study was that most of the study participants were women. One of the reasons was the low number of men going to health centers in Iran, but due to the clinical and health importance of the issue for women compared to men and more self-care of this group of people, this limitation can be ignored. Another’s limitation of this research is having a study design that assessed changed only, having few clusters, and sample size not powered to assess efficacy/effectiveness of intervention. Finally, other clinical parameters such as laboratory investigations were not measured in this study, as it would provide additional findings.

## Conclusion

This current study provided a basis to examine in the effectiveness of such intervention using a properly powered randomized controlled study. The theory of self-efficacy serves as an effective framework for modifying educational interventions for promoting preventive behavior in older adults. Therefore, healthcare providers can use these interventions to design appropriate interventions for older adults and improve their nutritional status.

## Data Availability

Datasets used and/or analyzed during the current study are available from the corresponding author on reasonable request.
